# 2-Oxo-2-(2-oxo-2*H*-chromen-3-yl)ethyl pyrrolidine-1-carbodi­thio­ate

**DOI:** 10.1107/S1600536813020965

**Published:** 2013-08-03

**Authors:** K. Mahesh Kumar, N. M. Mahabhaleshwaraiah, O. Kotresh, K. R. Roopashree, H. C. Devarajegowda

**Affiliations:** a Department of Chemistry, Karnatak University’s Karnatak Science College, Dharwad, Karnataka 580 001, India; b Department of Physics, Yuvaraja’s College (Constituent College), University of Mysore, Mysore 570 005, Karnataka, India

## Abstract

There are two independent mol­ecules in the asymmetric unit of the title compound, C_16_H_15_NO_3_S_2_, in which the pyrrolidine rings adopt envelope conformations, with a methyl­ene C atom as the flap. The dihedral angles betweeen the near-planar 2*H*-chromene ring systems [maximum deviations = 0.0167 (20) and 0.0136 (19) Å] and the pyrrolidine rings (all atoms) are 83.83 (11) and 82.43 (11)°. In the crystal, inversion dimers linked by pairs of C—H⋯O hydrogen bonds occur for one of the mol­ecules. Further C—H⋯O links involving both mol­ecules generate a three-dimensional network.

## Related literature

For a related structure and the synthesis of the title compound, see: Mahabaleshwaraiah *et al.* (2012 ▶).
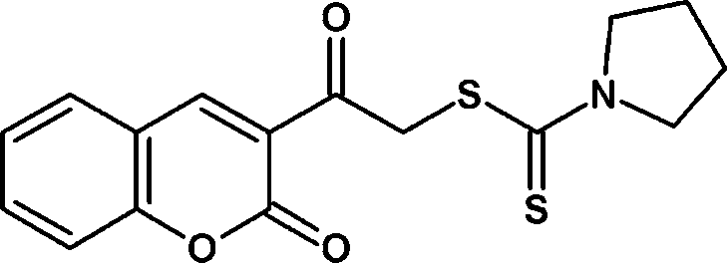


## Experimental

### 

#### Crystal data

C_16_H_15_NO_3_S_2_
*M* *_r_* = 333.41
Triclinic, 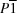

*a* = 9.7158 (2) Å
*b* = 12.5040 (2) Å
*c* = 13.5925 (2) Å
α = 106.415 (1)°
β = 100.882 (1)°
γ = 94.854 (1)°
*V* = 1538.74 (5) Å^3^
*Z* = 4
Mo *K*α radiation
μ = 0.36 mm^−1^
*T* = 296 K
0.24 × 0.20 × 0.12 mm

#### Data collection

Bruker SMART CCD diffractometer
Absorption correction: multi-scan (*SADABS*; Bruker, 2001 ▶) *T*_min_ = 0.770, *T*_max_ = 1.000
21575 measured reflections
5420 independent reflections
4477 reflections with *I* > 2σ(*I*)
*R*_int_ = 0.023

#### Refinement

*R*[*F* ^2^ > 2σ(*F* ^2^)] = 0.039
*wR*(*F* ^2^) = 0.109
*S* = 1.09
5420 reflections
397 parameters
H-atom parameters constrained
Δρ_max_ = 0.34 e Å^−3^
Δρ_min_ = −0.38 e Å^−3^

### 

Data collection: *SMART* (Bruker, 2001 ▶); cell refinement: *SAINT* (Bruker, 2001 ▶); data reduction: *SAINT*; program(s) used to solve structure: *SHELXS97* (Sheldrick, 2008 ▶); program(s) used to refine structure: *SHELXL97* (Sheldrick, 2008 ▶); molecular graphics: *ORTEP-3 for Windows* (Farrugia, 2012 ▶); software used to prepare material for publication: *SHELXL97*.

## Supplementary Material

Crystal structure: contains datablock(s) I, global. DOI: 10.1107/S1600536813020965/hb7109sup1.cif

Structure factors: contains datablock(s) I. DOI: 10.1107/S1600536813020965/hb7109Isup2.hkl

Supplementary material file. DOI: 10.1107/S1600536813020965/hb7109Isup3.cml

Additional supplementary materials:  crystallographic information; 3D view; checkCIF report

## Figures and Tables

**Table 1:** Hydrogen-bond geometry (Å, °)

*D*—H⋯*A*	*D*—H	H⋯*A*	*D*⋯*A*	*D*—H⋯*A*
C9*A*—H9*A*⋯O5*B* ^i^	0.93	2.48	3.307 (2)	149
C15*A*—H15*A*⋯O5*B* ^i^	0.93	2.50	3.319 (3)	147
C9*B*—H9*B*⋯O5*A* ^i^	0.93	2.46	3.288 (3)	149
C15*B*—H15*B*⋯O5*A* ^i^	0.93	2.50	3.319 (3)	146
C17*B*—H17*B*⋯O3*B* ^ii^	0.97	2.47	3.432 (3)	170

Symmetry codes: (i) 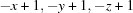; (ii) 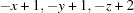.

**Fig. 1.**
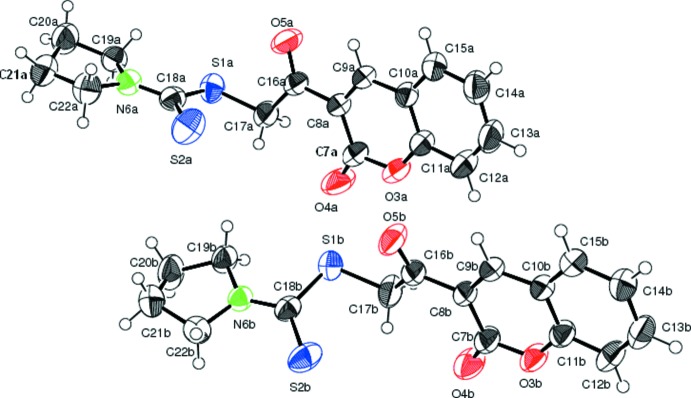
The molecular structure of the title compound. Displacement ellipsoids are drawn at the 50% probability level.

## supplementary crystallographic information

### 1. Comment

As part of our ongoing structural studies of coumarin derivatives with possible biological activity (Mahabaleshwaraiah *et al.*, 2012), we now describe the structure of the title compound, (I).

There are two independent molecules in the asymmetric unit of 2-oxo-2- (2-oxo-2*H*-chromen-3-yl)ethyl pyrrolidine-1-carbodithioate is shown in Fig. 1. The 2*H*-chromene ring systems (O3a/C7a–C15*a*) and (O3b/C7b–C15*b*) are nearly planar, with a maximum deviation of 0.0167 (20) Å and 0.0136 (19) Å respectively. The pyrrolidine rings (N6a/C19*a*—C22*a*) and (N6b/C19*b*—C22*b*) are not coplanar with the 2*H*-chromene ring systems (O3a/C7a–C15*a*) and (O3b/C7b–C15*b*); the dihedral angles between two planes being 83.83 (11)° and 82.43 (11)° in the two molecules. In the crystal, inversion related C17B—H17B···O3B hydrogen bonds generate *R*_2_^2^(12) loops.

### 2. Experimental

This compound was prepared according to the reported method (Mahabaleshwaraiah *et al.*, 2012). Colourless needles were grown from a mixed solution of EtOH/CHCl_3_ (v/v = 2/1) by slow evaporation at room temperature. Yield = 88%, m.p. 441 K.

### 3. Refinement

All H atoms were positioned geometrically, with C—H = 0.93 Å for aromatic H, C—H = 0.97 Å for methylene H and C—H = 0.96 Å for methyl H and refined using a riding model with *U*_iso_(H) = 1.5*U*_eq_(C) for methyl H and *U*_iso_(H) = 1.2*U*_eq_(C) for all other H.

### Crystal data

**Table:**

C_16_H_15_NO_3_S_2_	*Z* = 4
*M**_r_* = 333.41	*F*(000) = 696
Triclinic, *P*1	*D*_x_ = 1.439 Mg m^−^^3^
Hall symbol: -P 1	Melting point: 441 K
*a* = 9.7158 (2) Å	Mo *K*α radiation, λ = 0.71073 Å
*b* = 12.5040 (2) Å	Cell parameters from 5420 reflections
*c* = 13.5925 (2) Å	θ = 1.7–25.0°
α = 106.415 (1)°	µ = 0.36 mm^−^^1^
β = 100.882 (1)°	*T* = 296 K
γ = 94.854 (1)°	Plate, colourless
*V* = 1538.74 (5) Å^3^	0.24 × 0.20 × 0.12 mm

### Data collection

**Table:**

Bruker SMART CCD diffractometer	5420 independent reflections
Radiation source: fine-focus sealed tube	4477 reflections with *I* > 2σ(*I*)
Graphite monochromator	*R*_int_ = 0.023
ω and φ scans	θ_max_ = 25.0°, θ_min_ = 1.7°
Absorption correction: multi-scan (*SADABS*; Bruker, 2001)	*h* = −11→11
*T*_min_ = 0.770, *T*_max_ = 1.000	*k* = −14→14
21575 measured reflections	*l* = −16→16

### Refinement

**Table:**

Refinement on *F*^2^	Primary atom site location: structure-invariant direct methods
Least-squares matrix: full	Secondary atom site location: difference Fourier map
*R*[*F*^2^ > 2σ(*F*^2^)] = 0.039	Hydrogen site location: inferred from neighbouring sites
*wR*(*F*^2^) = 0.109	H-atom parameters constrained
*S* = 1.09	*w* = 1/[σ^2^(*F*_o_^2^) + (0.0528*P*)^2^ + 0.3725*P*] where *P* = (*F*_o_^2^ + 2*F*_c_^2^)/3
5420 reflections	(Δ/σ)_max_ = 0.001
397 parameters	Δρ_max_ = 0.34 e Å^−^^3^
0 restraints	Δρ_min_ = −0.38 e Å^−^^3^

### Special details

**Table:**

Experimental. IR (KBr): 636 cm^-1^(C—S), 1254 cm^-1^(C=S), 1070 cm^-1^(C—O), 854 cm^-1^ (C—N),1125 cm^-1^(C—O—C), 1694 cm^-1^ (C=O), 1731 cm^-1^(Coumarin C=O). GCMS: m/e: 335. 1H NMR (400 MHz, CDCl_3_, δ,. p.p.m): 2.11(m, 2H, Pyrrolidine-CH_2_) 2.16(m, 2H, Pyrrolidine-CH_2_), 3.68(t, 2H, Pyrrolidine-CH_2_), 3.91(t, 2H, Pyrrolidine-CH_2_),4.59(s, 2H, Methylene-CH_2_), 7.24(t, 1H, Ar—H), 7.41(d, 1H, Ar—H), 7.49(t, 1H, Ar—H), 7.65(d,1*H*, Ar—H),8.16(s, 1H, Ar—H). Elemental analysis for C_16_H_15_NO_3_S_2_: C, 57.58; H, 4.46; N, 4.13.
Geometry. All s.u.'s (except the s.u. in the dihedral angle between two l.s. planes) are estimated using the full covariance matrix. The cell s.u.'s are taken into account individually in the estimation of s.u.'s in distances, angles and torsion angles; correlations between s.u.'s in cell parameters are only used when they are defined by crystal symmetry. An approximate (isotropic) treatment of cell s.u.'s is used for estimating s.u.'s involving l.s. planes.
Refinement. Refinement of *F*^2^ against ALL reflections. The weighted *R*-factor *wR* and goodness of fit *S* are based on *F*^2^, conventional *R*-factors *R* are based on *F*, with *F* set to zero for negative *F*^2^. The threshold expression of *F*^2^ > 2σ(*F*^2^) is used only for calculating *R*-factors(gt) *etc*. and is not relevant to the choice of reflections for refinement. *R*-factors based on *F*^2^ are statistically about twice as large as those based on *F*, and *R*- factors based on ALL data will be even larger.

### Fractional atomic coordinates and isotropic or equivalent isotropic displacement parameters (Å^2^)

**Table:**

	*x*	*y*	*z*	*U*_iso_*/*U*_eq_	
S1B	0.46924 (6)	0.77672 (4)	0.81900 (5)	0.05885 (16)	
S2B	0.72066 (6)	0.92077 (6)	0.98610 (5)	0.07162 (19)	
O3B	0.74479 (16)	0.48705 (12)	1.07902 (11)	0.0598 (4)	
O4B	0.6049 (2)	0.61413 (15)	1.07471 (13)	0.0813 (5)	
O5B	0.68855 (18)	0.62686 (13)	0.78589 (12)	0.0704 (4)	
N6B	0.55263 (15)	0.98720 (12)	0.84624 (12)	0.0455 (4)	
C7B	0.6776 (2)	0.55533 (17)	1.02806 (16)	0.0566 (5)	
C8B	0.7036 (2)	0.54840 (15)	0.92381 (15)	0.0470 (4)	
C9B	0.7859 (2)	0.47448 (15)	0.88210 (15)	0.0474 (4)	
H9B	0.8008	0.4702	0.8156	0.057*	
C10B	0.85098 (19)	0.40263 (15)	0.93565 (15)	0.0461 (4)	
C11B	0.8291 (2)	0.41211 (16)	1.03604 (15)	0.0502 (5)	
C12B	0.8900 (2)	0.34839 (19)	1.09553 (18)	0.0627 (6)	
H12B	0.8739	0.3559	1.1623	0.075*	
C13B	0.9745 (2)	0.27392 (19)	1.05399 (19)	0.0652 (6)	
H13B	1.0165	0.2304	1.0931	0.078*	
C14B	0.9988 (2)	0.26217 (17)	0.95419 (18)	0.0612 (5)	
H14B	1.0565	0.2110	0.9272	0.073*	
C15B	0.9378 (2)	0.32573 (16)	0.89536 (16)	0.0549 (5)	
H15B	0.9543	0.3176	0.8286	0.066*	
C16B	0.6446 (2)	0.62295 (15)	0.86268 (16)	0.0513 (5)	
C17B	0.5292 (3)	0.68791 (18)	0.89563 (19)	0.0650 (6)	
H17A	0.5630	0.7342	0.9683	0.078*	
H17B	0.4489	0.6347	0.8928	0.078*	
C18B	0.58542 (19)	0.90472 (16)	0.88551 (15)	0.0464 (4)	
C19B	0.4308 (2)	0.97956 (16)	0.76104 (17)	0.0568 (5)	
H19A	0.3446	0.9454	0.7730	0.068*	
H19B	0.4458	0.9359	0.6936	0.068*	
C20B	0.4238 (3)	1.10145 (19)	0.7655 (2)	0.0776 (7)	
H20A	0.3921	1.1074	0.6954	0.093*	
H20B	0.3588	1.1328	0.8082	0.093*	
C21B	0.5699 (3)	1.16160 (18)	0.8132 (2)	0.0697 (6)	
H21A	0.5687	1.2402	0.8496	0.084*	
H21B	0.6245	1.1574	0.7597	0.084*	
C22B	0.6319 (2)	1.10183 (16)	0.88939 (17)	0.0573 (5)	
H22A	0.7323	1.1007	0.8926	0.069*	
H22B	0.6185	1.1381	0.9594	0.069*	
S1A	−0.04479 (6)	0.82131 (5)	0.38879 (5)	0.06212 (17)	
S2A	0.26976 (7)	0.90519 (6)	0.46861 (6)	0.0836 (2)	
O3A	0.24069 (18)	0.53930 (13)	0.65250 (11)	0.0711 (4)	
O4A	0.1038 (2)	0.66877 (16)	0.64994 (13)	0.0907 (6)	
O5A	0.0825 (2)	0.61136 (15)	0.32787 (13)	0.0935 (6)	
N6A	0.09101 (16)	0.98182 (13)	0.34318 (13)	0.0503 (4)	
C7A	0.1645 (3)	0.60259 (19)	0.59960 (17)	0.0620 (5)	
C8A	0.1661 (2)	0.57919 (16)	0.48808 (15)	0.0512 (5)	
C9A	0.2381 (2)	0.49904 (15)	0.44312 (15)	0.0518 (5)	
H9A	0.2358	0.4836	0.3717	0.062*	
C10A	0.3180 (2)	0.43666 (15)	0.50018 (16)	0.0507 (5)	
C11A	0.3162 (2)	0.45940 (17)	0.60620 (16)	0.0585 (5)	
C12A	0.3893 (3)	0.4023 (2)	0.6678 (2)	0.0774 (7)	
H12A	0.3854	0.4167	0.7381	0.093*	
C13A	0.4674 (3)	0.3242 (2)	0.6226 (2)	0.0787 (7)	
H13A	0.5183	0.2861	0.6634	0.094*	
C14A	0.4726 (3)	0.30032 (18)	0.5179 (2)	0.0698 (6)	
H14A	0.5268	0.2470	0.4891	0.084*	
C15A	0.3977 (2)	0.35547 (16)	0.45664 (18)	0.0598 (5)	
H15A	0.4001	0.3387	0.3859	0.072*	
C16A	0.0888 (2)	0.64165 (17)	0.42166 (16)	0.0582 (5)	
C17A	0.0179 (3)	0.73924 (18)	0.47142 (18)	0.0632 (6)	
H17C	0.0847	0.7885	0.5333	0.076*	
H17D	−0.0616	0.7101	0.4948	0.076*	
C18A	0.1125 (2)	0.91016 (16)	0.39811 (16)	0.0524 (5)	
C19A	−0.0455 (2)	0.99070 (17)	0.28054 (16)	0.0546 (5)	
H19C	−0.1066	1.0253	0.3253	0.065*	
H19D	−0.0935	0.9172	0.2351	0.065*	
C20A	−0.0040 (3)	1.0652 (2)	0.2166 (2)	0.0765 (7)	
H20C	0.0106	1.0197	0.1497	0.092*	
H20D	−0.0773	1.1107	0.2035	0.092*	
C21A	0.1297 (2)	1.1377 (2)	0.2816 (2)	0.0690 (6)	
H21C	0.1099	1.2057	0.3291	0.083*	
H21D	0.1877	1.1587	0.2373	0.083*	
C22A	0.2031 (2)	1.06729 (19)	0.3421 (2)	0.0672 (6)	
H22C	0.2740	1.0322	0.3078	0.081*	
H22D	0.2489	1.1131	0.4132	0.081*	

### Atomic displacement parameters (Å^2^)

**Table:**

	*U*^11^	*U*^22^	*U*^33^	*U*^12^	*U*^13^	*U*^23^
S1B	0.0681 (3)	0.0498 (3)	0.0632 (4)	0.0148 (2)	0.0129 (3)	0.0236 (2)
S2B	0.0666 (4)	0.0952 (4)	0.0564 (4)	0.0269 (3)	−0.0002 (3)	0.0330 (3)
O3B	0.0772 (9)	0.0722 (9)	0.0451 (8)	0.0197 (7)	0.0246 (7)	0.0319 (7)
O4B	0.1223 (14)	0.0904 (11)	0.0576 (10)	0.0506 (11)	0.0490 (10)	0.0349 (9)
O5B	0.1029 (12)	0.0805 (10)	0.0563 (9)	0.0452 (9)	0.0402 (9)	0.0419 (8)
N6B	0.0439 (8)	0.0489 (8)	0.0451 (9)	0.0114 (6)	0.0063 (7)	0.0177 (7)
C7B	0.0768 (14)	0.0566 (11)	0.0469 (12)	0.0160 (10)	0.0236 (10)	0.0238 (10)
C8B	0.0619 (11)	0.0454 (10)	0.0391 (10)	0.0085 (8)	0.0164 (9)	0.0181 (8)
C9B	0.0622 (11)	0.0472 (10)	0.0358 (10)	0.0058 (8)	0.0132 (8)	0.0168 (8)
C10B	0.0550 (10)	0.0449 (9)	0.0404 (10)	0.0045 (8)	0.0087 (8)	0.0180 (8)
C11B	0.0556 (11)	0.0541 (11)	0.0460 (11)	0.0041 (9)	0.0133 (9)	0.0233 (9)
C12B	0.0713 (13)	0.0773 (14)	0.0518 (13)	0.0088 (11)	0.0137 (10)	0.0398 (11)
C13B	0.0685 (13)	0.0698 (14)	0.0658 (15)	0.0113 (11)	0.0043 (11)	0.0407 (12)
C14B	0.0667 (13)	0.0572 (12)	0.0635 (14)	0.0165 (10)	0.0085 (11)	0.0260 (11)
C15B	0.0667 (12)	0.0539 (11)	0.0458 (12)	0.0113 (9)	0.0098 (9)	0.0191 (9)
C16B	0.0695 (12)	0.0454 (10)	0.0445 (11)	0.0122 (9)	0.0195 (10)	0.0168 (9)
C17B	0.0857 (15)	0.0598 (12)	0.0717 (15)	0.0276 (11)	0.0369 (12)	0.0374 (11)
C18B	0.0490 (10)	0.0569 (11)	0.0417 (10)	0.0206 (8)	0.0177 (8)	0.0195 (9)
C19B	0.0535 (11)	0.0582 (11)	0.0579 (13)	0.0092 (9)	−0.0022 (9)	0.0259 (10)
C20B	0.0760 (15)	0.0692 (14)	0.0923 (19)	0.0148 (12)	−0.0030 (13)	0.0449 (14)
C21B	0.0829 (16)	0.0533 (12)	0.0761 (16)	0.0101 (11)	0.0146 (13)	0.0267 (11)
C22B	0.0589 (12)	0.0525 (11)	0.0567 (13)	0.0043 (9)	0.0088 (10)	0.0146 (10)
S1A	0.0642 (3)	0.0604 (3)	0.0718 (4)	0.0186 (2)	0.0199 (3)	0.0301 (3)
S2A	0.0641 (4)	0.1074 (5)	0.0888 (5)	0.0289 (3)	0.0023 (3)	0.0495 (4)
O3A	0.1042 (12)	0.0781 (10)	0.0399 (8)	0.0224 (9)	0.0178 (8)	0.0279 (8)
O4A	0.1331 (16)	0.1087 (14)	0.0493 (10)	0.0566 (12)	0.0395 (10)	0.0302 (10)
O5A	0.1646 (18)	0.0908 (12)	0.0433 (10)	0.0714 (12)	0.0306 (10)	0.0290 (9)
N6A	0.0475 (8)	0.0547 (9)	0.0514 (10)	0.0140 (7)	0.0102 (7)	0.0191 (8)
C7A	0.0842 (15)	0.0650 (13)	0.0432 (12)	0.0146 (11)	0.0165 (11)	0.0240 (10)
C8A	0.0678 (12)	0.0491 (10)	0.0382 (11)	0.0061 (9)	0.0128 (9)	0.0161 (9)
C9A	0.0715 (13)	0.0500 (10)	0.0363 (10)	0.0061 (9)	0.0115 (9)	0.0183 (9)
C10A	0.0598 (11)	0.0456 (10)	0.0464 (11)	−0.0001 (8)	0.0059 (9)	0.0195 (9)
C11A	0.0740 (13)	0.0546 (11)	0.0458 (12)	0.0018 (10)	0.0046 (10)	0.0216 (10)
C12A	0.1071 (19)	0.0735 (15)	0.0520 (14)	0.0074 (14)	−0.0004 (13)	0.0332 (12)
C13A	0.0972 (18)	0.0650 (14)	0.0736 (18)	0.0093 (13)	−0.0082 (14)	0.0393 (13)
C14A	0.0748 (14)	0.0555 (12)	0.0796 (18)	0.0122 (10)	0.0044 (12)	0.0292 (12)
C15A	0.0723 (13)	0.0510 (11)	0.0575 (13)	0.0070 (10)	0.0085 (11)	0.0233 (10)
C16A	0.0842 (15)	0.0558 (11)	0.0418 (12)	0.0196 (10)	0.0203 (10)	0.0192 (9)
C17A	0.0853 (15)	0.0601 (12)	0.0555 (13)	0.0197 (11)	0.0271 (11)	0.0256 (11)
C18A	0.0589 (11)	0.0550 (11)	0.0463 (11)	0.0226 (9)	0.0147 (9)	0.0142 (9)
C19A	0.0530 (11)	0.0556 (11)	0.0536 (12)	0.0135 (9)	0.0046 (9)	0.0175 (10)
C20A	0.0835 (16)	0.0745 (15)	0.0727 (17)	0.0067 (12)	0.0010 (13)	0.0364 (13)
C21A	0.0718 (14)	0.0744 (14)	0.0741 (16)	0.0134 (11)	0.0256 (12)	0.0365 (13)
C22A	0.0547 (12)	0.0714 (14)	0.0800 (17)	0.0060 (10)	0.0163 (11)	0.0301 (12)

### Geometric parameters (Å, º)

**Table:**

S1B—C18B	1.773 (2)	S1A—C18A	1.774 (2)
S1B—C17B	1.787 (2)	S1A—C17A	1.785 (2)
S2B—C18B	1.6608 (19)	S2A—C18A	1.659 (2)
O3B—C11B	1.375 (2)	O3A—C11A	1.370 (3)
O3B—C7B	1.376 (2)	O3A—C7A	1.382 (2)
O4B—C7B	1.198 (2)	O4A—C7A	1.190 (3)
O5B—C16B	1.211 (2)	O5A—C16A	1.211 (2)
N6B—C18B	1.324 (2)	N6A—C18A	1.325 (2)
N6B—C19B	1.467 (2)	N6A—C22A	1.465 (3)
N6B—C22B	1.469 (2)	N6A—C19A	1.466 (2)
C7B—C8B	1.466 (3)	C7A—C8A	1.464 (3)
C8B—C9B	1.348 (3)	C8A—C9A	1.342 (3)
C8B—C16B	1.495 (3)	C8A—C16A	1.494 (3)
C9B—C10B	1.424 (2)	C9A—C10A	1.425 (3)
C9B—H9B	0.9300	C9A—H9A	0.9300
C10B—C11B	1.394 (3)	C10A—C11A	1.392 (3)
C10B—C15B	1.396 (3)	C10A—C15A	1.395 (3)
C11B—C12B	1.379 (3)	C11A—C12A	1.383 (3)
C12B—C13B	1.367 (3)	C12A—C13A	1.368 (4)
C12B—H12B	0.9300	C12A—H12A	0.9300
C13B—C14B	1.390 (3)	C13A—C14A	1.381 (4)
C13B—H13B	0.9300	C13A—H13A	0.9300
C14B—C15B	1.372 (3)	C14A—C15A	1.371 (3)
C14B—H14B	0.9300	C14A—H14A	0.9300
C15B—H15B	0.9300	C15A—H15A	0.9300
C16B—C17B	1.502 (3)	C16A—C17A	1.507 (3)
C17B—H17A	0.9700	C17A—H17C	0.9700
C17B—H17B	0.9700	C17A—H17D	0.9700
C19B—C20B	1.516 (3)	C19A—C20A	1.520 (3)
C19B—H19A	0.9700	C19A—H19C	0.9700
C19B—H19B	0.9700	C19A—H19D	0.9700
C20B—C21B	1.483 (3)	C20A—C21A	1.485 (3)
C20B—H20A	0.9700	C20A—H20C	0.9700
C20B—H20B	0.9700	C20A—H20D	0.9700
C21B—C22B	1.507 (3)	C21A—C22A	1.501 (3)
C21B—H21A	0.9700	C21A—H21C	0.9700
C21B—H21B	0.9700	C21A—H21D	0.9700
C22B—H22A	0.9700	C22A—H22C	0.9700
C22B—H22B	0.9700	C22A—H22D	0.9700
			
C18B—S1B—C17B	102.01 (11)	C18A—S1A—C17A	101.77 (10)
C11B—O3B—C7B	123.64 (15)	C11A—O3A—C7A	123.44 (16)
C18B—N6B—C19B	125.64 (16)	C18A—N6A—C22A	123.09 (17)
C18B—N6B—C22B	122.64 (16)	C18A—N6A—C19A	125.71 (17)
C19B—N6B—C22B	111.60 (14)	C22A—N6A—C19A	111.15 (16)
O4B—C7B—O3B	115.83 (18)	O4A—C7A—O3A	116.01 (19)
O4B—C7B—C8B	127.55 (19)	O4A—C7A—C8A	127.7 (2)
O3B—C7B—C8B	116.62 (17)	O3A—C7A—C8A	116.33 (19)
C9B—C8B—C7B	119.33 (17)	C9A—C8A—C7A	119.63 (18)
C9B—C8B—C16B	118.46 (16)	C9A—C8A—C16A	118.44 (17)
C7B—C8B—C16B	122.20 (17)	C7A—C8A—C16A	121.92 (18)
C8B—C9B—C10B	122.74 (17)	C8A—C9A—C10A	122.76 (18)
C8B—C9B—H9B	118.6	C8A—C9A—H9A	118.6
C10B—C9B—H9B	118.6	C10A—C9A—H9A	118.6
C11B—C10B—C15B	118.09 (17)	C11A—C10A—C15A	118.51 (19)
C11B—C10B—C9B	117.53 (17)	C11A—C10A—C9A	117.30 (19)
C15B—C10B—C9B	124.36 (17)	C15A—C10A—C9A	124.19 (19)
O3B—C11B—C12B	117.76 (18)	O3A—C11A—C12A	118.0 (2)
O3B—C11B—C10B	120.10 (16)	O3A—C11A—C10A	120.50 (18)
C12B—C11B—C10B	122.14 (19)	C12A—C11A—C10A	121.5 (2)
C13B—C12B—C11B	118.4 (2)	C13A—C12A—C11A	118.2 (2)
C13B—C12B—H12B	120.8	C13A—C12A—H12A	120.9
C11B—C12B—H12B	120.8	C11A—C12A—H12A	120.9
C12B—C13B—C14B	121.07 (19)	C12A—C13A—C14A	121.7 (2)
C12B—C13B—H13B	119.5	C12A—C13A—H13A	119.1
C14B—C13B—H13B	119.5	C14A—C13A—H13A	119.1
C15B—C14B—C13B	120.3 (2)	C15A—C14A—C13A	119.8 (2)
C15B—C14B—H14B	119.9	C15A—C14A—H14A	120.1
C13B—C14B—H14B	119.9	C13A—C14A—H14A	120.1
C14B—C15B—C10B	120.04 (19)	C14A—C15A—C10A	120.2 (2)
C14B—C15B—H15B	120.0	C14A—C15A—H15A	119.9
C10B—C15B—H15B	120.0	C10A—C15A—H15A	119.9
O5B—C16B—C8B	118.99 (17)	O5A—C16A—C8A	118.90 (18)
O5B—C16B—C17B	121.65 (18)	O5A—C16A—C17A	121.16 (19)
C8B—C16B—C17B	119.33 (17)	C8A—C16A—C17A	119.93 (17)
C16B—C17B—S1B	115.66 (15)	C16A—C17A—S1A	115.49 (15)
C16B—C17B—H17A	108.4	C16A—C17A—H17C	108.4
S1B—C17B—H17A	108.4	S1A—C17A—H17C	108.4
C16B—C17B—H17B	108.4	C16A—C17A—H17D	108.4
S1B—C17B—H17B	108.4	S1A—C17A—H17D	108.4
H17A—C17B—H17B	107.4	H17C—C17A—H17D	107.5
N6B—C18B—S2B	123.21 (15)	N6A—C18A—S2A	123.39 (16)
N6B—C18B—S1B	112.52 (14)	N6A—C18A—S1A	112.74 (14)
S2B—C18B—S1B	124.27 (11)	S2A—C18A—S1A	123.87 (12)
N6B—C19B—C20B	103.42 (16)	N6A—C19A—C20A	103.19 (17)
N6B—C19B—H19A	111.1	N6A—C19A—H19C	111.1
C20B—C19B—H19A	111.1	C20A—C19A—H19C	111.1
N6B—C19B—H19B	111.1	N6A—C19A—H19D	111.1
C20B—C19B—H19B	111.1	C20A—C19A—H19D	111.1
H19A—C19B—H19B	109.0	H19C—C19A—H19D	109.1
C21B—C20B—C19B	105.75 (17)	C21A—C20A—C19A	105.50 (19)
C21B—C20B—H20A	110.6	C21A—C20A—H20C	110.6
C19B—C20B—H20A	110.6	C19A—C20A—H20C	110.6
C21B—C20B—H20B	110.6	C21A—C20A—H20D	110.6
C19B—C20B—H20B	110.6	C19A—C20A—H20D	110.6
H20A—C20B—H20B	108.7	H20C—C20A—H20D	108.8
C20B—C21B—C22B	105.07 (18)	C20A—C21A—C22A	105.23 (18)
C20B—C21B—H21A	110.7	C20A—C21A—H21C	110.7
C22B—C21B—H21A	110.7	C22A—C21A—H21C	110.7
C20B—C21B—H21B	110.7	C20A—C21A—H21D	110.7
C22B—C21B—H21B	110.7	C22A—C21A—H21D	110.7
H21A—C21B—H21B	108.8	H21C—C21A—H21D	108.8
N6B—C22B—C21B	104.04 (16)	N6A—C22A—C21A	105.22 (17)
N6B—C22B—H22A	110.9	N6A—C22A—H22C	110.7
C21B—C22B—H22A	110.9	C21A—C22A—H22C	110.7
N6B—C22B—H22B	110.9	N6A—C22A—H22D	110.7
C21B—C22B—H22B	110.9	C21A—C22A—H22D	110.7
H22A—C22B—H22B	109.0	H22C—C22A—H22D	108.8
			
C11B—O3B—C7B—O4B	178.2 (2)	C11A—O3A—C7A—O4A	179.9 (2)
C11B—O3B—C7B—C8B	−2.1 (3)	C11A—O3A—C7A—C8A	0.8 (3)
O4B—C7B—C8B—C9B	−178.1 (2)	O4A—C7A—C8A—C9A	−178.3 (2)
O3B—C7B—C8B—C9B	2.3 (3)	O3A—C7A—C8A—C9A	0.6 (3)
O4B—C7B—C8B—C16B	3.5 (4)	O4A—C7A—C8A—C16A	1.3 (4)
O3B—C7B—C8B—C16B	−176.11 (17)	O3A—C7A—C8A—C16A	−179.78 (18)
C7B—C8B—C9B—C10B	−0.7 (3)	C7A—C8A—C9A—C10A	−1.9 (3)
C16B—C8B—C9B—C10B	177.76 (17)	C16A—C8A—C9A—C10A	178.45 (18)
C8B—C9B—C10B—C11B	−1.1 (3)	C8A—C9A—C10A—C11A	1.8 (3)
C8B—C9B—C10B—C15B	−179.45 (19)	C8A—C9A—C10A—C15A	−177.80 (19)
C7B—O3B—C11B—C12B	−179.84 (18)	C7A—O3A—C11A—C12A	179.4 (2)
C7B—O3B—C11B—C10B	0.3 (3)	C7A—O3A—C11A—C10A	−0.9 (3)
C15B—C10B—C11B—O3B	179.76 (17)	C15A—C10A—C11A—O3A	179.27 (18)
C9B—C10B—C11B—O3B	1.3 (3)	C9A—C10A—C11A—O3A	−0.4 (3)
C15B—C10B—C11B—C12B	0.0 (3)	C15A—C10A—C11A—C12A	−1.0 (3)
C9B—C10B—C11B—C12B	−178.46 (18)	C9A—C10A—C11A—C12A	179.3 (2)
O3B—C11B—C12B—C13B	−179.70 (19)	O3A—C11A—C12A—C13A	−178.6 (2)
C10B—C11B—C12B—C13B	0.1 (3)	C10A—C11A—C12A—C13A	1.7 (4)
C11B—C12B—C13B—C14B	−0.1 (3)	C11A—C12A—C13A—C14A	−1.1 (4)
C12B—C13B—C14B—C15B	0.1 (3)	C12A—C13A—C14A—C15A	−0.2 (4)
C13B—C14B—C15B—C10B	0.0 (3)	C13A—C14A—C15A—C10A	0.9 (3)
C11B—C10B—C15B—C14B	0.0 (3)	C11A—C10A—C15A—C14A	−0.3 (3)
C9B—C10B—C15B—C14B	178.29 (18)	C9A—C10A—C15A—C14A	179.32 (19)
C9B—C8B—C16B—O5B	−10.7 (3)	C9A—C8A—C16A—O5A	6.7 (3)
C7B—C8B—C16B—O5B	167.7 (2)	C7A—C8A—C16A—O5A	−172.9 (2)
C9B—C8B—C16B—C17B	167.16 (19)	C9A—C8A—C16A—C17A	−174.44 (19)
C7B—C8B—C16B—C17B	−14.4 (3)	C7A—C8A—C16A—C17A	6.0 (3)
O5B—C16B—C17B—S1B	−3.5 (3)	O5A—C16A—C17A—S1A	−11.3 (3)
C8B—C16B—C17B—S1B	178.68 (15)	C8A—C16A—C17A—S1A	169.88 (16)
C18B—S1B—C17B—C16B	−89.01 (19)	C18A—S1A—C17A—C16A	−80.90 (19)
C19B—N6B—C18B—S2B	−176.91 (15)	C22A—N6A—C18A—S2A	−2.4 (3)
C22B—N6B—C18B—S2B	−1.1 (3)	C19A—N6A—C18A—S2A	−179.34 (15)
C19B—N6B—C18B—S1B	3.0 (2)	C22A—N6A—C18A—S1A	177.18 (16)
C22B—N6B—C18B—S1B	178.87 (14)	C19A—N6A—C18A—S1A	0.2 (2)
C17B—S1B—C18B—N6B	−175.02 (14)	C17A—S1A—C18A—N6A	−178.87 (15)
C17B—S1B—C18B—S2B	4.90 (15)	C17A—S1A—C18A—S2A	0.69 (16)
C18B—N6B—C19B—C20B	167.00 (19)	C18A—N6A—C19A—C20A	−167.79 (19)
C22B—N6B—C19B—C20B	−9.2 (2)	C22A—N6A—C19A—C20A	15.0 (2)
N6B—C19B—C20B—C21B	25.7 (3)	N6A—C19A—C20A—C21A	−28.4 (2)
C19B—C20B—C21B—C22B	−32.7 (3)	C19A—C20A—C21A—C22A	31.4 (3)
C18B—N6B—C22B—C21B	173.23 (18)	C18A—N6A—C22A—C21A	−173.35 (19)
C19B—N6B—C22B—C21B	−10.4 (2)	C19A—N6A—C22A—C21A	4.0 (2)
C20B—C21B—C22B—N6B	26.3 (2)	C20A—C21A—C22A—N6A	−21.9 (3)

### Hydrogen-bond geometry (Å, º)

**Table:**

*D*—H···*A*	*D*—H	H···*A*	*D*···*A*	*D*—H···*A*
C9*A*—H9*A*···O5*B*^i^	0.93	2.48	3.307 (2)	149
C15*A*—H15*A*···O5*B*^i^	0.93	2.50	3.319 (3)	147
C9*B*—H9*B*···O5*A*^i^	0.93	2.46	3.288 (3)	149
C15*B*—H15*B*···O5*A*^i^	0.93	2.50	3.319 (3)	146
C17*B*—H17*B*···O3*B*^ii^	0.97	2.47	3.432 (3)	170

Symmetry codes: (i) −*x*+1, −*y*+1, −*z*+1; (ii) −*x*+1, −*y*+1, −*z*+2.

## References

[bb1] Bruker (2001). *SMART*, *SAINT* and *SADABS* Bruker AXS Inc., Madison,Wisconsin, USA.

[bb2] Farrugia, L. J. (2012). *J. Appl. Cryst.* **45**, 849–854.

[bb3] Mahabaleshwaraiah, N. M., Kumar, K. M., Kotresh, O., Al-eryani, W. F. A. & Devarajegowda, H. C. (2012). *Acta Cryst.* E**68**, o1566. 10.1107/S1600536812017953 PMC3344662 22590424

[bb4] Sheldrick, G. M. (2008). *Acta Cryst.* A**64**, 112–122. 10.1107/S0108767307043930 18156677

